# Crystal structure and Hirshfeld surface analysis of 2,2′-[(3,5-di-*tert*-butyl-4-hy­droxy­phen­yl)methanedi­yl]bis­(3-hy­droxy-5,5-di­methyl­cyclo­hex-2-en-1-one)

**DOI:** 10.1107/S2056989023003171

**Published:** 2023-04-14

**Authors:** Ali N. Khalilov, Victor N. Khrustalev, Larissa V. Aleksandrova, Mehmet Akkurt, Rovnag M. Rzayev, Ajaya Bhattarai, İbrahim G. Mamedov

**Affiliations:** a "Composite Materials" Scientific Research Center, Azerbaijan State Economic University (UNEC), H. Aliyev str. 135, Az 1063, Baku, Azerbaijan; b Department of Chemistry, Baku State University, Z. Khalilov str. 23, Az, 1148, Baku, Azerbaijan; c Peoples’ Friendship University of Russia (RUDN University), Miklukho-Maklay St.6, Moscow, 117198, Russian Federation; d N. D. Zelinsky Institute of Organic Chemistry RAS, Leninsky Prosp. 47, Moscow, 119991, Russian Federation; e Department of Physics, Faculty of Sciences, Erciyes University, 38039 Kayseri, Türkiye; f Department of Chemistry, M.M.A.M.C (Tribhuvan University) Biratnagar, Nepal; Venezuelan Institute of Scientific Research, Venezuela

**Keywords:** crystal structure, hydrogen bonds, hydrogen-bonded zigzag chains, van der Waals inter­actions, 1,8-dioxo-octa­hydroxanthene, Hirshfeld surface analysis

## Abstract

In the title compound, C_31_H_44_O_5_, mol­ecules are connected by O—H⋯O and C—H⋯O hydrogen bonds, forming hydrogen-bonded zigzag chains running along the *b* axis and parallel to the (001) plane. The mol­ecular packing is stabilized by van der Waals inter­actions between these chains along the *a* and *c* axes. The inter­molecular inter­actions in the crystal structure were qu­anti­fied and analysed using Hirshfeld surface analysis.

## Abstract 2

In the title compound, mol­ecules are linked together by O—H⋯O and C—H⋯O hydrogen bonds, forming zigzag chains that are parallel to the (001) plane and run along the *b*-axis direction. van der Waals inter­actions between these chains along the *a* and *c* axes maintain the mol­ecular packing.

## 1. Chemical context

The various carbon–carbon bond-formation techniques play important roles in organic chemistry (Celik *et al.*, 2023 ▸; Chalkha *et al.*, 2023 ▸; Tapera *et al.*, 2022 ▸). Xanthene derivatives have broad applications in medicine as a result of their anti-inflammatory, anti­bacterial, anti­viral, anti­fungal, anti-depressant, anti­plasmodial and anti-malarial activity (Maia *et al.*, 2021 ▸). They are a special class of oxygen-incorporating tricyclic systems. The xanthene moiety is also found in various natural compounds and has a wide spectrum of therapeutic and pharmacological properties. Aside from medicinal applications, xanthene dyes have been used for diagnostic and imaging applications (Khan & Sekar, 2022 ▸; Majumdar *et al.*, 2022 ▸; Lakhrissi *et al.*, 2022 ▸). 
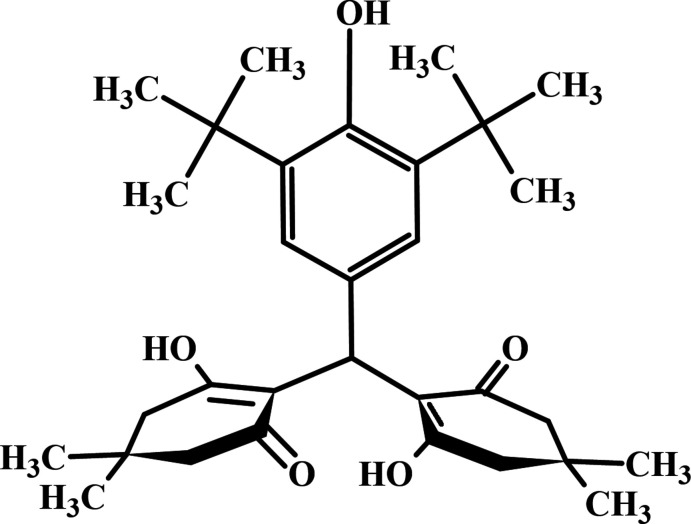


Thus, in the framework of our ongoing structural studies (Zubkov *et al.*, 2018 ▸; Gurbanov *et al.*, 2020 ▸; Maharramov *et al.*, 2021 ▸, 2022 ▸), we report the crystal structure and Hirshfeld surface analysis of the title compound, 2,2′-[(3,5-di-*tert*-butyl-4-hy­droxy­phen­yl)methanedi­yl]bis­(3-hy­droxy-5,5-di­methyl­cyclo­hex-2-en-1-one).

## 2. Structural commentary

**Figure 1:**
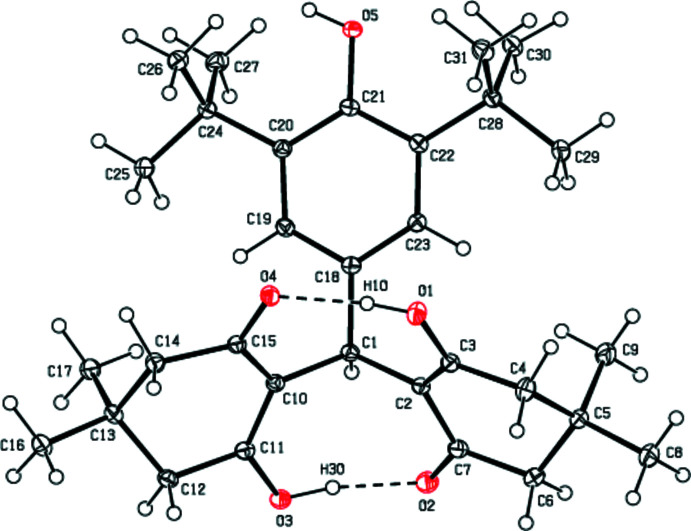
The mol­ecular structure of the title compound, showing the atom labelling and displacement ellipsoids drawn at the 30% probability level.

As seen in Fig. 1 ▸, each of the cyclo­hexenone rings (C2–C7 and C10–C15) of the title compound adopts an envelope conformation. The puckering parameters (Cremer & Pople, 1975 ▸) are *Q*_T_ = 0.5027 (12) Å, θ = 63.26 (14)°, φ = 179.78 (16)° for the C2–C7 ring, and *Q*_T_ = 0.4920 (11) Å, θ = 67.89 (13)°, φ = 167.63 (14)° for the C10–C15 ring. The mean planes [maximum deviations are 0.353 (1) Å for C5 and 0.332 (1) Å for C13] of the cyclo­hexane rings C2–C7 and C10–C15 subtend a dihedral angle of 39.59 (5)°, and they form dihedral angles of 56.25 (5) and 50.23 (5)°, respectively, with the benzene ring (C18–C23) of the 3,5-di-tert-butyl-4-hy­droxy­phenyl moiety. The bond lengths and angles in the title compound are within normal ranges. The orientation of the hy­droxy and carbonyl O atoms permits the formation of two intra­molecular O—H⋯O hydrogen bonds as they face one another (Fig. 1 ▸, Table 1 ▸).

## 3. Supra­molecular features and Hirshfeld surface analysis

**Figure 2:**
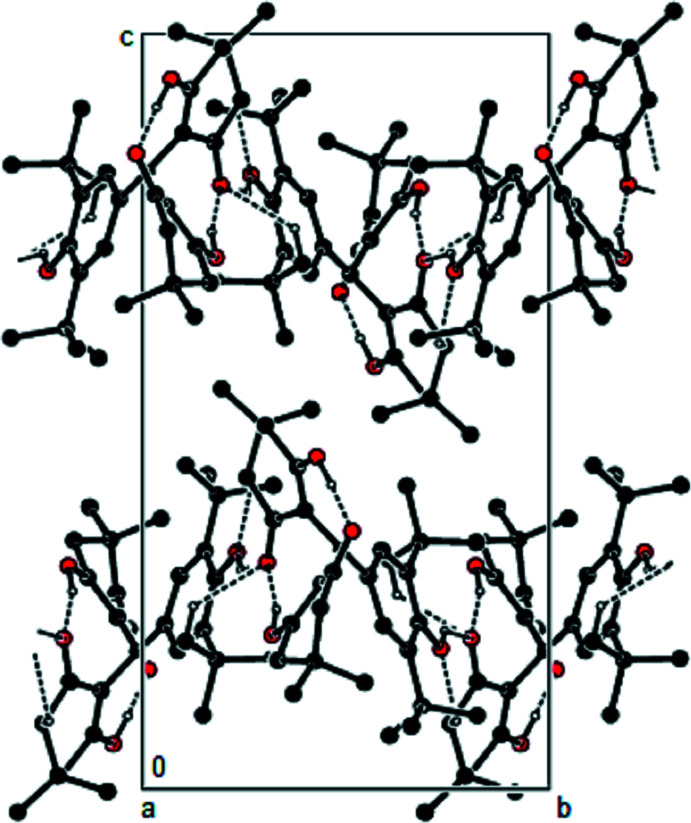
The packing of the title compound viewed along the *a*-axis with O—H⋯O and C—H⋯O hydrogen bonds shown as dashed lines.

In the crystal, O—H⋯O and C—H⋯O hydrogen bonds (Table 1 ▸) link the mol­ecules, forming zigzag chains running along the [010] direction and parallel to the (001) plane (Figs. 2 ▸ and 3 ▸). The mol­ecular packing is stabilized by van der Waals inter­actions between these chains along the *a* and *c* axes.

**Figure 4:**
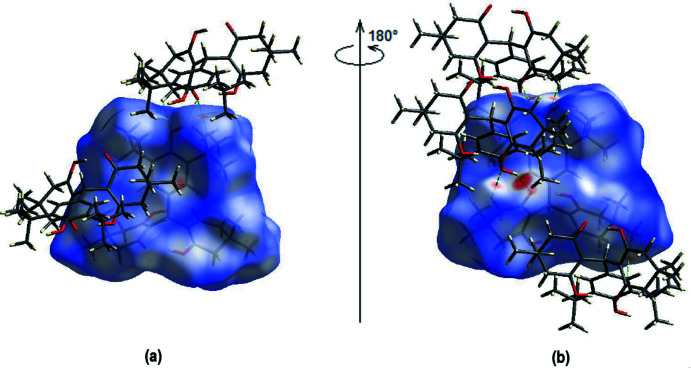
(*a*) Front and (*b*) back sides of the three-dimensional Hirshfeld surface of the title compound mapped over *d*_norm_, with a fixed colour scale of −0.4467 to 1.6498 a.u.

To qu­antify the inter­molecular inter­actions, a Hirshfeld surface analysis was performed and *CrystalExplorer17* (Turner *et al.*, 2017 ▸) was used to obtain the accompanying two-dimensional fingerprint plots. Fig. 4 ▸ shows the Hirshfeld surface mapped onto *d*_norm_ using a common surface resolution and a constant color scale of −0.4467 (red) to 1.6498 (blue) a.u. On the Hirshfeld surface, shorter and longer contacts are indicated by red and blue spots, respectively, and contacts with lengths about equal to the sum of the van der Waals radii are indicated by white spots. The O—H⋯O and C—H⋯O inter­actions are represented by the two most significant red spots on the *d*_norm_ surface (Tables 1 ▸ and 2 ▸).

**Figure 5:**
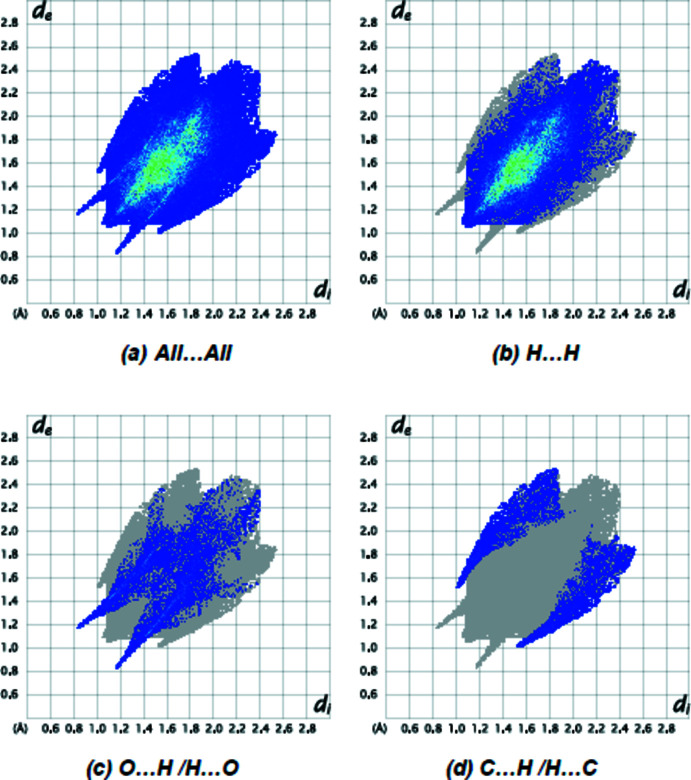
The two-dimensional fingerprint plots of the title compound, showing (*a*) all inter­actions, and delineated into (*b*) H⋯H, (*c*) O⋯H/H⋯O and (*d*) C⋯H/H⋯C inter­actions. [*d*_e_ and *d*_i_ represent the distances from a point on the Hirshfeld surface to the nearest atoms outside (external) and inside (inter­nal) the surface, respectively.]

Fig. 5 ▸ depicts the two-dimensional fingerprint plots of (*d*_i_, *d*_e_) points from all the contacts contributing to the Hirshfeld surface analysis in normal mode for all atoms. The most important inter­molecular inter­actions are H⋯H contacts, contributing 76.8% to the overall crystal packing. Other inter­actions and their respective contributions are O⋯H/H⋯O (15.2%), C⋯H/H⋯C (6.9%) and O⋯O (1.0%). The Hirshfeld surface study verifies the significance of H-atom inter­actions in the packing formation. The significant frequency of H⋯H and O⋯H/H⋯O inter­actions implies that van der Waals inter­actions and hydrogen bonding are important in crystal packing (Hathwar *et al.*, 2015 ▸).

## 4. Database survey

The ten most similar compounds found in a search of the Cambridge Structural Database (CSD, Version 5.42, update of September 2021; Groom *et al.*, 2016 ▸) for the 2,2′-(ethane-1,1-di­yl)bis­(3-hy­droxy-5,5-di­methyl­cyclo­hex-2-en-1-one) moiety are 2,2′-[(4-eth­oxy­phen­yl)methyl­ene]bis­(3-hy­droxy-5,5-di­methyl­cyclo­hex-2-en-1- one) (**I**; Sureshbabu & Sughanya, 2012 ▸), 2,2′-[(3-bromo-4-hy­droxy-5-meth­oxy­phen­yl)methyl­idene]bis­(3-hy­droxy-5,5-di­methyl­cyclo­hex-2-en-1-one) (**II**; Sughanya & Sureshbabu, 2012 ▸), 2,2′-[(1*E*)-3-phenyl­prop-2-ene-1,1-di­yl]bis­(3-hy­droxy-5,5-di­methyl­cyclo­hex-2-en-1-one) (**III**; Zhu *et al.*, 2011 ▸), (*E*)-2,2′-[3-(4-chloro­phen­yl)prop-2-ene-1,1-di­yl]bis­(3-hy­droxy-5,5-di­methyl­cyclo­hex-2-en-1-one) (**IV**; Cha *et al.*, 2013*a* ▸), (*E*)-2,2′-[3-(4-fluoro­phen­yl)prop-2-ene-1,1-di­yl]bis­(3-hy­droxy-5,5-di­methyl­cyclo­hex-2-en-1-one) (**V**; Cha *et al.*, 2013*b* ▸), (*E*)-2,2′-[3-(2-nitro­phen­yl)prop-2-ene-1,1-di­yl]bis­(3-hy­droxy-5,5-di­methyl­cyclo­hex- 2-en-1-one) (**VI**; Cha *et al.*, 2011 ▸), 2,2′-[(*E*)-3-(4-nitro­phen­yl)prop-2-ene-1,1-di­yl]bis­(3-hy­droxy-5,5-di­methyl­cyclo­hex-2-en-1-one) (**VII**; Cha *et al.*, 2012 ▸), bis­(2- hy­droxy-4,4-dimethyl-6-oxo-1-cyclo­hex­en­yl)phenyl­methane (**VIII**; Bolte *et al.*, 1997*a* ▸), 2,2′-[(2-nitro­phen­yl)methyl­ene]bis­(3-hy­droxy-5,5-di­methyl­cyclo­hex-2-en­one) (**IX**; Steiger *et al.*, 2020 ▸) and 2,2′-[(3-hy­droxy­phen­yl)methyl­ene]bis­(3-hy­droxy-5,5-dimethyl-2-cyclo­hexen-1-one) (**X**; Bolte *et al.*, 2001*b* ▸).

In **I**, **II**, **III**, **IV**, **VIII**, **IX** and **X**, the two cyclo­hexane rings adopt an envelope conformation, while in **VI** and **VII** they exhibit a half-chair conformation. In all of these crystals, mol­ecules are connected *via* O—H⋯O hydrogen bonds. In **X**, there are also O—H⋯O hydrogen bonds involving the water mol­ecules. In **III**, **IV**, **V**, **VI**, **VII** and **IX**, C—H⋯O hydrogen bonds also contribute to the cohesion of the crystal structure.

## 5. Synthesis and crystallization

To a solution of 3,5-di-*tert*-butyl-4-hy­droxy­benzaldehyde (1 g, 4.3 mmol) and 5,5-di­methyl­cyclo­hexane-1,3-dione (1.2 g, 8.6 mmol) in ethanol (15 mL), piperidine (2–3 drops) was added and the mixture was refluxed for 3 h. Then 10 mL of ethanol was removed from the reaction mixture, which was left overnight. The precipitated crystals were separated by filtration and recrystallized from an ethanol/water (4:1) solution (yield 65%; m.p. 465–466 K).

^1^H NMR (300 MHz, CDCl_3_, ppm): 1.05 (*s*, 6H, 2CH_3_), 1.08 (*s*, 6H, 2CH_3_), 1.41 (*s*, 18H, 6CH_3_), 2.05–2.35 (*m*, 8H, 4CH_2_), 5.39 (*s*, 1H, CH), 5.69 (*s*, 1H, OH), 6.65 (*s*, 2H, arom.), 11.21 (*s*, 2H, 2OH); ^13^C NMR (75 MHz, CDCl_3_, ppm): 26.4, 28.7, 30.8, 31.7, 32.6, 36.5, 45.3, 51.8, 111.6, 122.9, 13.8, 136.8, 153.2, 176.4, 202.3.

## 6. Refinement

Crystal data, data collection and structure refinement details are summarized in Table 3 ▸. All C-bound H atoms were placed at calculated positions and refined using a riding model, with C—H = 0.95–1.00 Å, and with *U*_iso_(H) = 1.2 or 1.5*U*_eq_(C). The O-bound H atoms were located in a difference-Fourier map and were freely refined.

## Supplementary Material

Crystal structure: contains datablock(s) I. DOI: 10.1107/S2056989023003171/zn2027sup1.cif

Structure factors: contains datablock(s) I. DOI: 10.1107/S2056989023003171/zn2027Isup2.hkl

Supporting information file. DOI: 10.1107/S2056989023003171/zn2027Isup3.cml

CCDC reference: 2254247

Additional supporting information:  crystallographic information; 3D view; checkCIF report

## Figures and Tables

**Figure 3:**
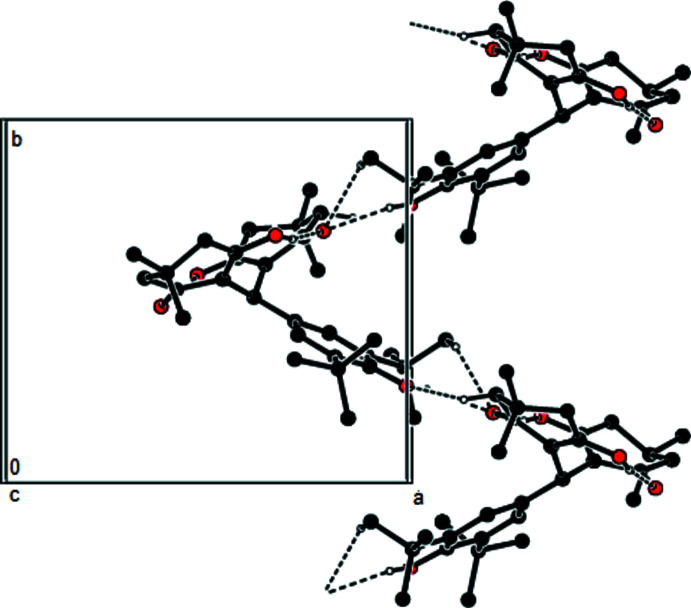
A view of the zigzag chains running along the *b*-axis direction of the title compound with O—H⋯O and C—H⋯O hydrogen bonds shown as dashed lines.

**Table 1:** Hydrogen-bond geometry (Å, °)

*D*—H⋯*A*	*D*—H	H⋯*A*	*D*⋯*A*	*D*—H⋯*A*
O1—H1*O*⋯O4	0.930 (19)	1.711 (19)	2.6201 (11)	164.7 (17)
O3—H3*O*⋯O2	0.950 (19)	1.68 (2)	2.6174 (11)	170.3 (17)
O5—H5*O*⋯O4^i^	0.848 (19)	2.128 (18)	2.8285 (11)	139.7 (16)
C14—H14*A*⋯O5^ii^	0.99	2.48	3.1912 (12)	128

Symmetry codes: (i) 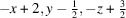 ; (ii) 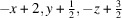 .

**Table 2:** Summary of short inter­atomic contacts (Å) in the title compound

H4*B*⋯H16*B*	2.39	*x*, 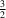 − *y*, 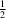 + *z*
H4*A*⋯H1	2.31	1 − *x*, 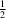 + *y*, 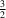 − *z*
H17*B*⋯O2	2.65	1 − *x*, 1 − *y*, 1 − *z*
O4⋯H5*O*	2.12	2 − *x*, 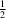 + *y*, 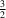 − *z*
C17⋯H30*B*	3.10	*x*, 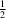 − *y*, − 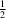 + *z*
H26*C*⋯H6*A*	2.58	1 + *x*, *y*, *z*
H25*B*⋯H17*A*	2.57	2 − *x*, 1 − *y*, 1 − *z*

**Table 3:** Experimental details

Crystal data	
Chemical formula	C_31_H_44_O_5_
*M*_r_	496.66
Crystal system, space group	Monoclinic, *P*2_1_/*c*
Temperature (K)	100
*a*, *b*, *c* (Å)	12.40591 (9), 10.98934 (10), 20.58063 (17)
β (°)	98.4293 (7)
*V* (Å^3^)	2775.50 (4)
*Z*	4
Radiation type	Cu *K*α
μ (mm^−1^)	0.63
Crystal size (mm)	0.33 × 0.21 × 0.18

Data collection	
Diffractometer	XtaLAB Synergy, Dualflex, HyPix
Absorption correction	Gaussian (*CrysAlis PRO*; Rigaku OD, 2022 ▸)
*T*_min_, *T*_max_	0.362, 1.000
No. of measured, independent and observed [*I* > 2σ(*I*)] reflections	61789, 5870, 5540
*R*_int_	0.047
(sin θ/λ)_max_ (Å^−1^)	0.634

Refinement	
*R*[*F* ^2^ > 2σ(*F* ^2^)], *wR*(*F* ^2^), *S*	0.039, 0.104, 1.03
No. of reflections	5870
No. of parameters	347
H-atom treatment	H atoms treated by a mixture of independent and constrained refinement
Δρ_max_, Δρ_min_ (e Å^−3^)	0.27, −0.26

Computer programs: *CrysAlis PRO* (Rigaku OD, 2022 ▸), *SHELXT2014/5* (Sheldrick, 2015*a* ▸), *SHELXL2018/3* (Sheldrick, 2015*b* ▸), *ORTEP-3 for Windows* (Farrugia, 2012 ▸) and *PLATON* (Spek, 2020 ▸).

## supplementary crystallographic information

### Crystal data

**Table:**

C_31_H_44_O_5_	*F*(000) = 1080
*M**_r_* = 496.66	*D*_x_ = 1.189 Mg m^−^^3^
Monoclinic, *P*2_1_/*c*	Cu *K*α radiation, λ = 1.54184 Å
*a* = 12.40591 (9) Å	Cell parameters from 41183 reflections
*b* = 10.98934 (10) Å	θ = 3.6–77.8°
*c* = 20.58063 (17) Å	µ = 0.63 mm^−^^1^
β = 98.4293 (7)°	*T* = 100 K
*V* = 2775.50 (4) Å^3^	Prism, colourless
*Z* = 4	0.33 × 0.21 × 0.18 mm

### Data collection

**Table:**

XtaLAB Synergy, Dualflex, HyPix diffractometer	5540 reflections with *I* > 2σ(*I*)
Radiation source: micro-focus sealed X-ray tube	*R*_int_ = 0.047
φ and ω scans	θ_max_ = 77.9°, θ_min_ = 3.6°
Absorption correction: gaussian (CrysAlisPro; Rigaku OD, 2022)	*h* = −13→15
*T*_min_ = 0.362, *T*_max_ = 1.000	*k* = −13→13
61789 measured reflections	*l* = −26→26
5870 independent reflections	

### Refinement

**Table:**

Refinement on *F*^2^	Primary atom site location: difference Fourier map
Least-squares matrix: full	Secondary atom site location: difference Fourier map
*R*[*F*^2^ > 2σ(*F*^2^)] = 0.039	Hydrogen site location: mixed
*wR*(*F*^2^) = 0.104	H atoms treated by a mixture of independent and constrained refinement
*S* = 1.03	*w* = 1/[σ^2^(*F*_o_^2^) + (0.0581*P*)^2^ + 0.9674*P*] where *P* = (*F*_o_^2^ + 2*F*_c_^2^)/3
5870 reflections	(Δ/σ)_max_ = 0.001
347 parameters	Δρ_max_ = 0.27 e Å^−^^3^
0 restraints	Δρ_min_ = −0.26 e Å^−^^3^

### Special details

**Table:**

Geometry. All esds (except the esd in the dihedral angle between two l.s. planes) are estimated using the full covariance matrix. The cell esds are taken into account individually in the estimation of esds in distances, angles and torsion angles; correlations between esds in cell parameters are only used when they are defined by crystal symmetry. An approximate (isotropic) treatment of cell esds is used for estimating esds involving l.s. planes.

### Fractional atomic coordinates and isotropic or equivalent isotropic displacement parameters (Å^2^)

**Table:**

	*x*	*y*	*z*	*U*_iso_*/*U*_eq_	
O1	0.67352 (6)	0.68092 (7)	0.79581 (4)	0.02223 (17)	
H1O	0.7151 (15)	0.6706 (16)	0.7621 (9)	0.046 (5)*	
O2	0.39003 (6)	0.48506 (8)	0.65828 (4)	0.02641 (18)	
O3	0.47791 (6)	0.57142 (7)	0.55974 (4)	0.02175 (17)	
H3O	0.4532 (15)	0.5356 (17)	0.5969 (9)	0.049 (5)*	
O4	0.79022 (6)	0.69306 (7)	0.69970 (3)	0.01951 (16)	
O5	0.99582 (6)	0.26695 (8)	0.81386 (4)	0.02279 (17)	
H5O	1.0464 (15)	0.2572 (16)	0.7907 (9)	0.041 (4)*	
C1	0.61903 (8)	0.51072 (9)	0.68241 (5)	0.0160 (2)	
H1	0.5758	0.4455	0.6566	0.019*	
C2	0.54278 (8)	0.56043 (9)	0.72776 (5)	0.0171 (2)	
C3	0.57463 (8)	0.63356 (9)	0.78073 (5)	0.0186 (2)	
C4	0.50033 (9)	0.66692 (11)	0.82932 (6)	0.0245 (2)	
H4A	0.4701	0.7491	0.8186	0.029*	
H4B	0.5435	0.6707	0.8737	0.029*	
C5	0.40583 (9)	0.57748 (10)	0.83053 (5)	0.0217 (2)	
C6	0.35058 (9)	0.56319 (12)	0.75965 (6)	0.0265 (2)	
H6A	0.2947	0.4985	0.7579	0.032*	
H6B	0.3127	0.6401	0.7454	0.032*	
C7	0.42799 (9)	0.53188 (10)	0.71194 (5)	0.0211 (2)	
C8	0.32572 (10)	0.62886 (12)	0.87359 (6)	0.0307 (3)	
H8A	0.3632	0.6401	0.9185	0.046*	
H8B	0.2650	0.5719	0.8739	0.046*	
H8C	0.2977	0.7073	0.8559	0.046*	
C9	0.44652 (10)	0.45389 (12)	0.85835 (6)	0.0304 (3)	
H9A	0.4965	0.4189	0.8307	0.046*	
H9B	0.3844	0.3991	0.8590	0.046*	
H9C	0.4848	0.4646	0.9031	0.046*	
C10	0.64598 (8)	0.60075 (9)	0.63062 (5)	0.0162 (2)	
C11	0.57732 (8)	0.61877 (9)	0.57293 (5)	0.0176 (2)	
C12	0.60567 (8)	0.69745 (10)	0.51805 (5)	0.0197 (2)	
H12A	0.5757	0.7800	0.5224	0.024*	
H12B	0.5709	0.6634	0.4756	0.024*	
C13	0.72887 (8)	0.70697 (10)	0.51767 (5)	0.0192 (2)	
C14	0.78192 (8)	0.74319 (10)	0.58701 (5)	0.0193 (2)	
H14A	0.8614	0.7292	0.5905	0.023*	
H14B	0.7707	0.8315	0.5926	0.023*	
C15	0.74074 (8)	0.67709 (9)	0.64281 (5)	0.0169 (2)	
C16	0.75463 (10)	0.80530 (11)	0.46958 (6)	0.0266 (2)	
H16A	0.7221	0.8826	0.4804	0.040*	
H16B	0.7244	0.7814	0.4247	0.040*	
H16C	0.8338	0.8148	0.4728	0.040*	
C17	0.77368 (9)	0.58492 (10)	0.49695 (5)	0.0231 (2)	
H17A	0.8532	0.5899	0.5002	0.035*	
H17B	0.7422	0.5667	0.4515	0.035*	
H17C	0.7543	0.5202	0.5259	0.035*	
C18	0.72031 (8)	0.44419 (9)	0.71819 (5)	0.0169 (2)	
C19	0.80443 (8)	0.41235 (9)	0.68346 (5)	0.0176 (2)	
H19	0.7982	0.4341	0.6384	0.021*	
C20	0.89755 (8)	0.34983 (9)	0.71204 (5)	0.0174 (2)	
C21	0.90520 (8)	0.32072 (9)	0.77939 (5)	0.0177 (2)	
C22	0.82019 (8)	0.34622 (9)	0.81580 (5)	0.0175 (2)	
C23	0.72883 (8)	0.40755 (9)	0.78330 (5)	0.0177 (2)	
H23	0.6702	0.4248	0.8068	0.021*	
C24	0.98864 (9)	0.31798 (10)	0.67141 (5)	0.0206 (2)	
C25	0.95624 (10)	0.35071 (13)	0.59845 (5)	0.0307 (3)	
H25A	0.8885	0.3087	0.5811	0.046*	
H25B	1.0142	0.3255	0.5738	0.046*	
H25C	0.9454	0.4388	0.5941	0.046*	
C26	1.09148 (9)	0.39337 (11)	0.69613 (6)	0.0249 (2)	
H26A	1.0742	0.4803	0.6916	0.037*	
H26B	1.1491	0.3734	0.6700	0.037*	
H26C	1.1164	0.3743	0.7424	0.037*	
C27	1.01253 (9)	0.17986 (11)	0.67306 (6)	0.0249 (2)	
H27A	1.0304	0.1529	0.7188	0.037*	
H27B	1.0742	0.1632	0.6496	0.037*	
H27C	0.9481	0.1359	0.6519	0.037*	
C28	0.82892 (9)	0.31386 (10)	0.88945 (5)	0.0190 (2)	
C29	0.72191 (9)	0.33977 (11)	0.91629 (5)	0.0248 (2)	
H29A	0.7048	0.4267	0.9118	0.037*	
H29B	0.7299	0.3167	0.9628	0.037*	
H29C	0.6628	0.2924	0.8914	0.037*	
C30	0.85384 (9)	0.17769 (10)	0.90225 (5)	0.0221 (2)	
H30A	0.7966	0.1284	0.8769	0.033*	
H30B	0.8564	0.1604	0.9492	0.033*	
H30C	0.9244	0.1578	0.8888	0.033*	
C31	0.91838 (9)	0.39252 (10)	0.92908 (5)	0.0235 (2)	
H31A	0.9879	0.3783	0.9130	0.035*	
H31B	0.9254	0.3705	0.9757	0.035*	
H31C	0.8987	0.4787	0.9237	0.035*	

### Atomic displacement parameters (Å^2^)

**Table:**

	*U*^11^	*U*^22^	*U*^33^	*U*^12^	*U*^13^	*U*^23^
O1	0.0206 (4)	0.0269 (4)	0.0198 (4)	−0.0059 (3)	0.0049 (3)	−0.0048 (3)
O2	0.0183 (4)	0.0367 (5)	0.0231 (4)	−0.0052 (3)	−0.0008 (3)	−0.0003 (3)
O3	0.0160 (4)	0.0296 (4)	0.0183 (4)	−0.0029 (3)	−0.0017 (3)	−0.0008 (3)
O4	0.0183 (4)	0.0242 (4)	0.0154 (3)	−0.0040 (3)	0.0005 (3)	−0.0014 (3)
O5	0.0179 (4)	0.0335 (4)	0.0168 (3)	0.0085 (3)	0.0021 (3)	0.0044 (3)
C1	0.0152 (5)	0.0165 (5)	0.0156 (4)	−0.0006 (4)	0.0002 (3)	−0.0001 (4)
C2	0.0156 (5)	0.0178 (5)	0.0178 (5)	0.0016 (4)	0.0017 (4)	0.0031 (4)
C3	0.0178 (5)	0.0184 (5)	0.0196 (5)	0.0007 (4)	0.0030 (4)	0.0029 (4)
C4	0.0252 (6)	0.0245 (5)	0.0254 (5)	−0.0009 (4)	0.0093 (4)	−0.0035 (4)
C5	0.0180 (5)	0.0250 (5)	0.0231 (5)	0.0034 (4)	0.0061 (4)	0.0048 (4)
C6	0.0157 (5)	0.0384 (7)	0.0255 (6)	0.0018 (4)	0.0034 (4)	0.0043 (5)
C7	0.0175 (5)	0.0236 (5)	0.0215 (5)	0.0004 (4)	0.0008 (4)	0.0044 (4)
C8	0.0269 (6)	0.0366 (7)	0.0312 (6)	0.0074 (5)	0.0129 (5)	0.0055 (5)
C9	0.0268 (6)	0.0315 (6)	0.0352 (6)	0.0062 (5)	0.0124 (5)	0.0131 (5)
C10	0.0162 (5)	0.0165 (5)	0.0157 (4)	0.0008 (4)	0.0015 (4)	−0.0007 (3)
C11	0.0169 (5)	0.0182 (5)	0.0173 (5)	0.0016 (4)	0.0009 (4)	−0.0022 (4)
C12	0.0198 (5)	0.0221 (5)	0.0161 (5)	0.0021 (4)	−0.0007 (4)	0.0009 (4)
C13	0.0203 (5)	0.0225 (5)	0.0145 (5)	0.0002 (4)	0.0015 (4)	0.0016 (4)
C14	0.0191 (5)	0.0210 (5)	0.0177 (5)	−0.0030 (4)	0.0022 (4)	0.0002 (4)
C15	0.0165 (5)	0.0180 (5)	0.0159 (4)	0.0018 (4)	0.0016 (4)	−0.0009 (4)
C16	0.0274 (6)	0.0315 (6)	0.0210 (5)	−0.0006 (5)	0.0033 (4)	0.0075 (4)
C17	0.0234 (5)	0.0273 (6)	0.0185 (5)	0.0028 (4)	0.0033 (4)	−0.0015 (4)
C18	0.0160 (5)	0.0163 (5)	0.0177 (5)	0.0002 (4)	0.0005 (4)	0.0001 (4)
C19	0.0189 (5)	0.0190 (5)	0.0147 (4)	0.0002 (4)	0.0015 (4)	0.0004 (4)
C20	0.0165 (5)	0.0188 (5)	0.0166 (5)	0.0005 (4)	0.0017 (4)	−0.0007 (4)
C21	0.0167 (5)	0.0184 (5)	0.0170 (5)	0.0018 (4)	−0.0003 (4)	0.0011 (4)
C22	0.0193 (5)	0.0175 (5)	0.0155 (5)	0.0004 (4)	0.0017 (4)	0.0007 (4)
C23	0.0168 (5)	0.0183 (5)	0.0182 (5)	0.0012 (4)	0.0036 (4)	0.0004 (4)
C24	0.0178 (5)	0.0278 (6)	0.0164 (5)	0.0046 (4)	0.0032 (4)	0.0019 (4)
C25	0.0249 (6)	0.0499 (8)	0.0182 (5)	0.0127 (5)	0.0067 (4)	0.0051 (5)
C26	0.0192 (5)	0.0281 (6)	0.0279 (5)	0.0018 (4)	0.0048 (4)	0.0071 (4)
C27	0.0223 (5)	0.0286 (6)	0.0235 (5)	0.0050 (4)	0.0024 (4)	−0.0053 (4)
C28	0.0211 (5)	0.0209 (5)	0.0151 (5)	0.0025 (4)	0.0029 (4)	0.0012 (4)
C29	0.0258 (6)	0.0315 (6)	0.0184 (5)	0.0060 (4)	0.0072 (4)	0.0039 (4)
C30	0.0265 (5)	0.0211 (5)	0.0186 (5)	0.0018 (4)	0.0028 (4)	0.0031 (4)
C31	0.0272 (6)	0.0237 (5)	0.0189 (5)	0.0006 (4)	0.0011 (4)	−0.0018 (4)

### Geometric parameters (Å, º)

**Table:**

O1—C3	1.3268 (13)	C14—H14B	0.9900
O1—H1O	0.930 (19)	C16—H16A	0.9800
O2—C7	1.2466 (14)	C16—H16B	0.9800
O3—C11	1.3293 (13)	C16—H16C	0.9800
O3—H3O	0.950 (19)	C17—H17A	0.9800
O4—C15	1.2521 (12)	C17—H17B	0.9800
O5—C21	1.3720 (12)	C17—H17C	0.9800
O5—H5O	0.848 (19)	C18—C23	1.3882 (14)
C1—C2	1.5241 (14)	C18—C19	1.3931 (14)
C1—C10	1.5270 (14)	C19—C20	1.3977 (14)
C1—C18	1.5442 (13)	C19—H19	0.9500
C1—H1	1.0000	C20—C21	1.4121 (14)
C2—C3	1.3654 (15)	C20—C24	1.5414 (14)
C2—C7	1.4479 (14)	C21—C22	1.4085 (14)
C3—C4	1.5015 (14)	C22—C23	1.4014 (14)
C4—C5	1.5329 (15)	C22—C28	1.5453 (13)
C4—H4A	0.9900	C23—H23	0.9500
C4—H4B	0.9900	C24—C25	1.5390 (14)
C5—C6	1.5262 (16)	C24—C26	1.5436 (16)
C5—C9	1.5305 (15)	C24—C27	1.5459 (16)
C5—C8	1.5329 (15)	C25—H25A	0.9800
C6—C7	1.5104 (15)	C25—H25B	0.9800
C6—H6A	0.9900	C25—H25C	0.9800
C6—H6B	0.9900	C26—H26A	0.9800
C8—H8A	0.9800	C26—H26B	0.9800
C8—H8B	0.9800	C26—H26C	0.9800
C8—H8C	0.9800	C27—H27A	0.9800
C9—H9A	0.9800	C27—H27B	0.9800
C9—H9B	0.9800	C27—H27C	0.9800
C9—H9C	0.9800	C28—C29	1.5373 (14)
C10—C11	1.3703 (14)	C28—C31	1.5423 (15)
C10—C15	1.4361 (14)	C28—C30	1.5429 (14)
C11—C12	1.5044 (14)	C29—H29A	0.9800
C12—C13	1.5332 (14)	C29—H29B	0.9800
C12—H12A	0.9900	C29—H29C	0.9800
C12—H12B	0.9900	C30—H30A	0.9800
C13—C16	1.5305 (15)	C30—H30B	0.9800
C13—C14	1.5338 (14)	C30—H30C	0.9800
C13—C17	1.5360 (15)	C31—H31A	0.9800
C14—C15	1.5098 (14)	C31—H31B	0.9800
C14—H14A	0.9900	C31—H31C	0.9800
			
C3—O1—H1O	111.9 (11)	H16A—C16—H16B	109.5
C11—O3—H3O	113.4 (11)	C13—C16—H16C	109.5
C21—O5—H5O	112.4 (12)	H16A—C16—H16C	109.5
C2—C1—C10	114.48 (8)	H16B—C16—H16C	109.5
C2—C1—C18	114.35 (8)	C13—C17—H17A	109.5
C10—C1—C18	113.17 (8)	C13—C17—H17B	109.5
C2—C1—H1	104.4	H17A—C17—H17B	109.5
C10—C1—H1	104.4	C13—C17—H17C	109.5
C18—C1—H1	104.4	H17A—C17—H17C	109.5
C3—C2—C7	117.84 (9)	H17B—C17—H17C	109.5
C3—C2—C1	124.54 (9)	C23—C18—C19	117.75 (9)
C7—C2—C1	117.58 (9)	C23—C18—C1	122.62 (9)
O1—C3—C2	124.54 (9)	C19—C18—C1	119.51 (9)
O1—C3—C4	112.67 (9)	C18—C19—C20	122.87 (9)
C2—C3—C4	122.78 (10)	C18—C19—H19	118.6
C3—C4—C5	113.58 (9)	C20—C19—H19	118.6
C3—C4—H4A	108.9	C19—C20—C21	117.18 (9)
C5—C4—H4A	108.9	C19—C20—C24	120.65 (9)
C3—C4—H4B	108.9	C21—C20—C24	122.14 (9)
C5—C4—H4B	108.9	O5—C21—C22	115.54 (9)
H4A—C4—H4B	107.7	O5—C21—C20	122.49 (9)
C6—C5—C9	110.09 (10)	C22—C21—C20	121.96 (9)
C6—C5—C4	106.73 (9)	C23—C22—C21	117.25 (9)
C9—C5—C4	111.35 (9)	C23—C22—C28	120.95 (9)
C6—C5—C8	110.61 (9)	C21—C22—C28	121.73 (9)
C9—C5—C8	108.47 (9)	C18—C23—C22	122.82 (9)
C4—C5—C8	109.60 (10)	C18—C23—H23	118.6
C7—C6—C5	113.92 (9)	C22—C23—H23	118.6
C7—C6—H6A	108.8	C25—C24—C20	111.64 (9)
C5—C6—H6A	108.8	C25—C24—C26	106.33 (9)
C7—C6—H6B	108.8	C20—C24—C26	109.60 (9)
C5—C6—H6B	108.8	C25—C24—C27	105.83 (9)
H6A—C6—H6B	107.7	C20—C24—C27	111.38 (9)
O2—C7—C2	121.33 (10)	C26—C24—C27	111.91 (9)
O2—C7—C6	118.45 (10)	C24—C25—H25A	109.5
C2—C7—C6	120.18 (10)	C24—C25—H25B	109.5
C5—C8—H8A	109.5	H25A—C25—H25B	109.5
C5—C8—H8B	109.5	C24—C25—H25C	109.5
H8A—C8—H8B	109.5	H25A—C25—H25C	109.5
C5—C8—H8C	109.5	H25B—C25—H25C	109.5
H8A—C8—H8C	109.5	C24—C26—H26A	109.5
H8B—C8—H8C	109.5	C24—C26—H26B	109.5
C5—C9—H9A	109.5	H26A—C26—H26B	109.5
C5—C9—H9B	109.5	C24—C26—H26C	109.5
H9A—C9—H9B	109.5	H26A—C26—H26C	109.5
C5—C9—H9C	109.5	H26B—C26—H26C	109.5
H9A—C9—H9C	109.5	C24—C27—H27A	109.5
H9B—C9—H9C	109.5	C24—C27—H27B	109.5
C11—C10—C15	117.13 (9)	H27A—C27—H27B	109.5
C11—C10—C1	121.77 (9)	C24—C27—H27C	109.5
C15—C10—C1	120.93 (8)	H27A—C27—H27C	109.5
O3—C11—C10	124.11 (9)	H27B—C27—H27C	109.5
O3—C11—C12	112.55 (8)	C29—C28—C31	107.37 (9)
C10—C11—C12	123.32 (9)	C29—C28—C30	106.28 (9)
C11—C12—C13	112.71 (8)	C31—C28—C30	110.04 (9)
C11—C12—H12A	109.1	C29—C28—C22	111.71 (8)
C13—C12—H12A	109.0	C31—C28—C22	109.34 (8)
C11—C12—H12B	109.0	C30—C28—C22	111.97 (8)
C13—C12—H12B	109.1	C28—C29—H29A	109.5
H12A—C12—H12B	107.8	C28—C29—H29B	109.5
C16—C13—C12	110.75 (9)	H29A—C29—H29B	109.5
C16—C13—C14	108.46 (9)	C28—C29—H29C	109.5
C12—C13—C14	107.76 (8)	H29A—C29—H29C	109.5
C16—C13—C17	108.58 (9)	H29B—C29—H29C	109.5
C12—C13—C17	110.09 (9)	C28—C30—H30A	109.5
C14—C13—C17	111.20 (8)	C28—C30—H30B	109.5
C15—C14—C13	115.84 (9)	H30A—C30—H30B	109.5
C15—C14—H14A	108.3	C28—C30—H30C	109.5
C13—C14—H14A	108.3	H30A—C30—H30C	109.5
C15—C14—H14B	108.3	H30B—C30—H30C	109.5
C13—C14—H14B	108.3	C28—C31—H31A	109.5
H14A—C14—H14B	107.4	C28—C31—H31B	109.5
O4—C15—C10	121.45 (9)	H31A—C31—H31B	109.5
O4—C15—C14	118.02 (9)	C28—C31—H31C	109.5
C10—C15—C14	120.51 (9)	H31A—C31—H31C	109.5
C13—C16—H16A	109.5	H31B—C31—H31C	109.5
C13—C16—H16B	109.5		
			
C10—C1—C2—C3	−80.31 (12)	C11—C10—C15—O4	159.09 (10)
C18—C1—C2—C3	52.59 (13)	C1—C10—C15—O4	−16.20 (15)
C10—C1—C2—C7	97.16 (11)	C11—C10—C15—C14	−19.37 (14)
C18—C1—C2—C7	−129.95 (9)	C1—C10—C15—C14	165.34 (9)
C7—C2—C3—O1	−170.29 (10)	C13—C14—C15—O4	171.74 (9)
C1—C2—C3—O1	7.17 (16)	C13—C14—C15—C10	−9.75 (14)
C7—C2—C3—C4	10.96 (15)	C2—C1—C18—C23	14.42 (14)
C1—C2—C3—C4	−171.58 (9)	C10—C1—C18—C23	147.93 (10)
O1—C3—C4—C5	−156.69 (9)	C2—C1—C18—C19	−169.61 (9)
C2—C3—C4—C5	22.20 (15)	C10—C1—C18—C19	−36.10 (13)
C3—C4—C5—C6	−51.77 (12)	C23—C18—C19—C20	−2.46 (15)
C3—C4—C5—C9	68.40 (12)	C1—C18—C19—C20	−178.62 (9)
C3—C4—C5—C8	−171.58 (9)	C18—C19—C20—C21	−1.08 (15)
C9—C5—C6—C7	−69.22 (12)	C18—C19—C20—C24	−179.42 (10)
C4—C5—C6—C7	51.76 (13)	C19—C20—C21—O5	−175.48 (9)
C8—C5—C6—C7	170.91 (10)	C24—C20—C21—O5	2.84 (16)
C3—C2—C7—O2	166.72 (10)	C19—C20—C21—C22	3.91 (15)
C1—C2—C7—O2	−10.92 (15)	C24—C20—C21—C22	−177.77 (10)
C3—C2—C7—C6	−10.86 (15)	O5—C21—C22—C23	176.38 (9)
C1—C2—C7—C6	171.50 (9)	C20—C21—C22—C23	−3.06 (15)
C5—C6—C7—O2	159.86 (10)	O5—C21—C22—C28	−0.72 (14)
C5—C6—C7—C2	−22.50 (15)	C20—C21—C22—C28	179.85 (9)
C2—C1—C10—C11	−82.41 (12)	C19—C18—C23—C22	3.40 (15)
C18—C1—C10—C11	144.14 (9)	C1—C18—C23—C22	179.43 (9)
C2—C1—C10—C15	92.66 (11)	C21—C22—C23—C18	−0.72 (15)
C18—C1—C10—C15	−40.79 (12)	C28—C22—C23—C18	176.40 (10)
C15—C10—C11—O3	−167.64 (9)	C19—C20—C24—C25	−6.12 (15)
C1—C10—C11—O3	7.61 (15)	C21—C20—C24—C25	175.62 (10)
C15—C10—C11—C12	10.77 (15)	C19—C20—C24—C26	111.43 (11)
C1—C10—C11—C12	−173.97 (9)	C21—C20—C24—C26	−66.83 (13)
O3—C11—C12—C13	−155.18 (9)	C19—C20—C24—C27	−124.18 (10)
C10—C11—C12—C13	26.24 (14)	C21—C20—C24—C27	57.56 (13)
C11—C12—C13—C16	−169.61 (9)	C23—C22—C28—C29	8.36 (14)
C11—C12—C13—C14	−51.12 (11)	C21—C22—C28—C29	−174.66 (10)
C11—C12—C13—C17	70.30 (11)	C23—C22—C28—C31	−110.35 (11)
C16—C13—C14—C15	163.76 (9)	C21—C22—C28—C31	66.63 (12)
C12—C13—C14—C15	43.82 (12)	C23—C22—C28—C30	127.43 (10)
C17—C13—C14—C15	−76.92 (11)	C21—C22—C28—C30	−55.59 (13)

### Hydrogen-bond geometry (Å, º)

**Table:**

*D*—H···*A*	*D*—H	H···*A*	*D*···*A*	*D*—H···*A*
O1—H1*O*···O4	0.930 (19)	1.711 (19)	2.6201 (11)	164.7 (17)
O3—H3*O*···O2	0.950 (19)	1.68 (2)	2.6174 (11)	170.3 (17)
O5—H5*O*···O4^i^	0.848 (19)	2.128 (18)	2.8285 (11)	139.7 (16)
C14—H14*A*···O5^ii^	0.99	2.48	3.1912 (12)	128

Symmetry codes: (i) −*x*+2, *y*−1/2, −*z*+3/2; (ii) −*x*+2, *y*+1/2, −*z*+3/2.
